# Preventing the next 'SARS' - European healthcare workers' attitudes towards monitoring their health for the surveillance of newly emerging infections: qualitative study

**DOI:** 10.1186/1471-2458-11-541

**Published:** 2011-07-08

**Authors:** Adamma Aghaizu, Gillian Elam, Fortune Ncube, Gail Thomson, Emese Szilágyi, Tim Eckmanns, Garyphallia Poulakou, Mike Catchpole

**Affiliations:** 1 HIV/STI Department, Health Protection Services, Health Protection Agency, 61 Colindale Avenue, London NW9 5EQ, UK; 2 Centre for HIV and Sexual Health Research, University College London, WC1E 6BT, UK; 3 Centre for Emergency Preparedness and Response, Health Protection Agency, Porton Down, Salisbury SP4 0JG, UK; 4 Department of Hospital Epidemiology, National Centre for Epidemiology, 1097 Budapest, Gyali u. 2-6, Hungary; 5 Department for Infectious Disease Epidemiology, Robert Koch Institute, DGZ-Ring 1, 13086 Berlin, Germany; 6 Department of Internal Medicine, University General Hospital Attikon, National and Kapodistrian University, Athens Medical School, 124 62 Athens, Greece

## Abstract

**Background:**

Hospitals are often the epicentres of newly circulating infections. Healthcare workers (HCWs) are at high risk of acquiring infectious diseases and may be among the first to contract emerging infections. This study aims to explore European HCWs' perceptions and attitudes towards monitoring their absence and symptom reports for surveillance of newly circulating infections.

**Methods:**

A qualitative study with thematic analysis was conducted using focus group methodology. Forty-nine hospital-based HCWs from 12 hospitals were recruited to six focus groups; two each in England and Hungary and one each in Germany and Greece.

**Results:**

HCWs perceived risk factors for occupationally acquired infectious diseases to be 1.) exposure to patients with undiagnosed infections 2.) break-down in infection control procedures 3.) immuno-naïvety and 4.) symptomatic colleagues. They were concerned that a lack of monitoring and guidelines for infectious HCWs posed a risk to staff and patients and felt employers failed to take a positive interest in their health. Staffing demands and loss of income were noted as pressures to attend work when unwell. In the UK, Hungary and Greece participants felt monitoring staff absence and the routine disclosure of symptoms could be appropriate provided the effectiveness and efficiency of such a system were demonstrable. In Germany, legislation, privacy and confidentiality were identified as barriers.

All HCWs highlighted the need for knowledge and structural improvements for timelier recognition of emerging infections. These included increased suspicion and awareness among staff and standardised, homogenous absence reporting systems.

**Conclusions:**

Monitoring absence and infectious disease symptom reports among HCWs may be a feasible means of surveillance for emerging infections in some settings. A pre-requisite will be tackling the drivers for symptomatic HCWs to attend work.

## Background

In the aftermath of the recent global outbreaks of SARS and H1N1, public health professionals are evaluating the response and management of these in order to establish lessons learned for the development of future strategic response frameworks [1]. Key to improving the timely response to outbreaks of newly circulating infections is the early recognition of unusual illness and clusters of cases, whilst the numbers affected are low. To achieve this, current infectious disease surveillance and health systems need to be further developed and enhanced.

Healthcare workers (HCWs) are at the frontline of any public health outbreak response, and therefore among the most vulnerable to emerging infectious diseases (EIDs), evident by the extent of morbidity and mortality caused by SARS and other outbreaks [2-7]. In the past, hospitals have become amplifiers of newly emerging infections due to transmission from patients to HCWs, owing to ineffective infection control measures and failing to recognise a new pathogen [4,8]. HCWs may therefore be the first to experience secondary transmission of an EID, and consequently a population where clusters of cases are first to appear. Monitoring HCWs' health could provide an important opportunity to detect newly circulating infections earlier, enhancing current efforts for their identification and control.

The European Commission funded project REACT (Response to Emerging infectious diseases: Assessment and development of Core capacities and Tools) aims to improve and harmonise the response to public health threats throughout the European Union (EU) [9]. As part of this, a conceptual framework for a Europe-wide sentinel monitoring system for infectious diseases in HCWs is being developed. A number of studies have examined syndromic surveillance systems in hospital settings for timely recognition of nosocomial outbreaks in HCWs with positive results[5,10,11]. This study aims to explore the views and attitudes of European HCWs towards such surveillance systems, monitoring absence and symptom reports for the early detection of unusual illness and clusters of cases in previously healthy HCWs.

## Methods

### Recruitment, participants and setting

We used qualitative research methods to obtain a variety of attitudes, views and opinions and sought to bring together healthcare professionals from similar settings, sharing similar experiences, but from diverse cultural and professional backgrounds. From May to September 2010, we conducted six 60-90 minute focus group discussions in four European countries: two each in England and Hungary and one each in Greece and Germany. The research team, comprising of a group of international partners in the aforementioned countries [12], employed a targeted recruitment strategy to include a group of expert staff of hospitals they either were affiliated to or were particularly relevant to the study, such as those with large infectious disease departments. Purposive sampling and snowballing techniques were used to recruit hospital-based HCWs from diverse professions, departments and responsibilities. As the aim of the research was to explore a specific topic in the context of a diverse range of views, we did not sample a representative group of the healthcare worker or hospital populations. The study intends to provide the ground work for highlighting which factors could be issues in a representative and or broader population. GE, GT, TE, ES and GP identified and emailed potential participants and distributed information sheets. In total 49 staff from 12 hospitals participated and all participants had experience of working in other hospitals. A description of participating hospitals and participants is provided in table 1. AA, GE and FN prepared a protocol, topic guide and information leaflets about the study which were translated by an external company. GE, GT, TE, ES and GP facilitated the sessions in their respective countries. All participants provided written consent, and were informed that they could cease to participate at any time. They were assured their participation would remain confidential and their answers analysed anonymously.

**Table 1:** Participating hospital and healthcare workers' characteristics

Country	Focus group session	Hospital type and number	Staff types	Length of employment at hospital (years)
UK	Group 1	Urban teaching hospital n = 1	Ward manager	9
			Infection control nurse	12
			Directorate manager	30
			Clinical nurse specialist	6
			Ward manager	20
			Occupational health nurse	12
			Matron	21
	Group 2		Consultant in infectious diseases	3
			A&E nurse	10
			Sister	5
			Registrar	2
			Medical trainee	3
			A & E matron	-- *
			Outside contractor/payroll	-- *
Greece	Group 1	Urban teaching hospital n = 1	Nurse manager	4
			Nurse in infectious diseases	15
			Unit director of A & E	12
			Occupational health practitioner	9
			Professor of medicine	-- *
			Nurse	-- *
			Nurse	-- *
			Nurse in physiology	22
			Director of infectious disease unit	23
			Nurse	10
			Medical practitioner	5
			Infectious disease nurse	2
			Infectious disease physician	
			Infectious disease physician	
			Infectious disease physician	
			Infectious disease nurse	-- *
Germany	Group 1	Urban hospitals n = 3	Hospital hygienist	2
			Hospital hygienist	3
			Senior physician and hospital hygienist	3
Hungary	Group 1	Urban hospitals n = 6	Physician, hospital epidemiologist and head of hospital hygiene and epidemiology department	25
			Nurse specialist for infection control	27
			Infection control manager and head of sterilisation unit	5
			Hospital epidemiologist head of hospital hygiene and epidemiology department and public health inspector	30
			Hospital epidemiologist and head of hospital hygiene and epidemiology department	25
			Medical hospital epidemiologist head of hospital hygiene and epidemiology department	20
	Group 2	Urban Hospital n = 1	Consultant in internal medicine	26
			Registrar for internal medicine	0.75
			Intensive care nurse	15
			Infectious disease nurse	39
			Nurse in neurology department	21
			Nurse in neurology department	9
			Nurse in internal medicine department	14
			Nurse in internal medicine department	6
			Nurse in chronic/long term internal medicine	31
			Nurse in chronic/long term internal medicine	19

*missing data

To avoid potential response bias, where possible participants were split into two groups dividing senior from junior staff. In countries where only one session was held, the groups were structured such that either line managers or their staff and not both participated.

### Data collection and analysis

Each of the discussions was conducted in the primary language of the host country and audio recorded. Discussions were conducted with the aid of the topic guide for which facilitators in each country had received briefing via a teleconference. We used open ended questions intending the discussions to be participant led (examples given in appendix 1). The recordings were transcribed verbatim and back-translated into English by an external company. The transcripts were coded and analysed by two independent authors, AA and GE. Analytic themes were developed inductively in consultation with all authors and used to organise the data. A constant comparative method was used to consecutively inform sampling, topic guide development and analyses[13]. As the study sample is based on purely qualitative research methods, aiming solely to identify possible themes, we did not measure the frequency of responses or attempt to weight them. Finally, all recordings were destroyed after the transcripts and analyses were verified for accuracy.

### Ethical considerations

This study did not require ethical approval. An official waiver was granted from participating institutions.

## Results

Key findings from the discussions were HCWs' views on risk factors for occupationally acquired infectious diseases, their perceptions of illness and sickness-related absence, their views on monitoring absence and the structured collection of symptom data, factors affecting the willingness to report symptoms, factors affecting the validity of self-reported symptom data and knowledge and structural barriers for early recognition of nosocomial outbreaks.

### Healthcare workers' views on risk factors for occupationally acquired infections

All participants described experiences of nosocomial infectious disease transmission in terms of infections they had acquired or witnessed colleagues acquire, stating common infections to be gastrointestinal and respiratory viruses, and less common meningitis, hepatitis, TB and HIV. They identified risk factors for occupationally acquired infections to be (1) exposure to patients with undiagnosed infectious diseases, especially in accident and emergency wards, (2) break-down in infection control procedures due to lack of knowledge, resources and equipment, (3) immuno-naïvety and (4) symptomatic colleagues; all of which are likely to occur during an EID outbreak. In general, nosocomial disease transmission risk was perceived to be low, although UK participants considered healthy people to be more likely to contract infectious diseases in a hospital than in the community. A breakdown in infection control procedures was deemed acceptable in some situations as some participants in Hungary, who worked in intensive care departments, expressed the view that they have '*more pressing issues*' than following infection control procedures, such as '*saving lives*'. Other members of this group however felt that staff, in particular accident and emergency staff, needed to be '*more afraid of patients*' and thought intensive care staff to be more at risk, but also more aware. It was generally accepted that it was the responsibility of the individual HCW to report suspicion of nosocomial transmission incidences, though they expressed the belief that there was a need for more policies to protect the most vulnerable, such as for accident and emergency and pregnant staff. Participants' statements on risk factors for occupationally acquired infectious diseases are presented in appendix 2.

### Healthcare workers' perceptions of illness and sickness-related absence

Participants believed HCWs' health to be perceived of low importance by themselves, peers and employers, resulting in little sympathy towards their own health needs. It emerged that they often experienced anxiety when reporting absence from work with an illness and described common feelings of scepticism and distrust between themselves and line managers. Consequently, they were reluctant to stay at home with mild symptoms and even felt they were expected to come to work under such conditions to relieve managers from staffing pressures as, at times, managers were required to cover these shifts themselves. They felt they lacked understanding concerning the relationship between severity of symptoms and infectiousness and at which point they may pose a risk to others.

The participants commented on the varying illness behaviour patterns and absence reporting practices across professions with doctors being those most likely to attend work with infectious disease symptoms (appendix 3). They stated that only severe symptoms such as high fever and acute pain would encourage higher level staff to be absent and highlighted a lack of clear guidelines for symptomatic staff in the work place. In addition, in Hungary, staff sick leave entitlements amount to less than their actual pay, further motivating HCWs to come to work when unwell or take sick leave as annual leave. Participants' statements on their perceptions of illness and sickness-related absence are presented in appendix 3.

### Healthcare workers' views on monitoring their absence and symptoms for the surveillance of emerging infections and their willingness to report

During the discussions, a framework for a syndromic surveillance system was described to participants whereby absent staff, who suspected themselves of having a contagious illness, would be encouraged to report symptoms for daily monitoring and identification of potential outbreaks [12]. All felt they had experienced activities similar to those suggested, having been asked to report symptoms, stay away from work and in some cases provide specimens when unwell during the recent H1N1 outbreak.

Whilst all HCWs agreed colleagues habitually reported symptoms informally when notifying their absence, a number of benefits of frequent monitoring were highlighted such as the ability to 1.) identify staff absenteeism, 2.) improve infection control monitoring, and 3.) prevent spread of infections resulting in reduced rates of illness.

Willingness to disclose symptoms for routine surveillance however varied. UK participants were more open towards discussing symptoms and were of the view their colleagues would also be, in cases where the sickness was genuine and the rationale of the reporting system understood (appendix 4). They were nevertheless also concerned that detailed enquiries could be perceived as intrusive and provoke or increase feelings of anxiety and distrust. Further, they believed the sensitivities around disclosing this level of detail would vary depending on the individual, employer-employee relationships and the type of symptom, potentially undermining the validity of the data (appendix 4). Nevertheless, the concept was positively received as opportunities were identified for hospitals to give direction on how to manage symptomatic staff and, they in fact, anticipated reduced rates of absence.

In Hungary and Greece participants were also positive towards the concept of monitoring symptom reports and agreed this would lead to the earlier identification of outbreaks and improved management. However, they were sceptical as to how effective this would be for EIDs where the characteristics of the novel pathogen may be poorly understood.

In contrast, participants in Germany were less in favour of the suggestion that employers enquire about and formally record reported symptoms as part of absence data recording. They described the tight legislation around employer-employee relations, where currently it is unlawful for employers to request personal health details. They expressed the need for the protection of employee privacy and confidentiality and thought the risks associated with nosocomial transmission of EIDs were too small to compromise these rights. They were concerned about potential consequences for employment upon discovering an infectious HCW, and about the risk of victimisation, describing the existing controversy for hepatitis and HIV infected HCWs (appendix 4). However, they recognised a need for balancing staff and patient safety with confidentiality and the right to privacy, and identified situations where workers' councils needed to adopt a more proactive rather than reactive approach to identifying and preventing nosocomial transmission. HCWs' views on monitoring absence and symptom reports for surveillance of emerging infections are presented in appendix 4.

### Knowledge and structural barriers for the timely recognition of outbreaks among hospital staff

Beyond aspects relating to monitoring absence and symptom reports to detect outbreaks, participants in all groups voiced their concerns regarding local knowledge and structural barriers for earlier identification of EIDs.

Firstly, HCWs were concerned the lack of experience and low perceived risks of exposure to new pathogens would minimise awareness among hospital staff, leading to reduced suspicion and increased delay to the recognition of a new pathogen. Specialist input would be required for the monitoring process, introducing further labour and financial burdens on hospitals. In addition, the need for HCWs to be discriminating as to which symptoms to report, could result in wide variation in reporting practice, again potentially undermining the validity of the system. With EIDs viewed as rare events, participants felt that if the detection of EIDs was the sole rationale for reporting, adherence could decline as HCWs might question the usefulness of such a system.

Secondly, participants queried the heterogeneity in absence reporting across professions and working groups within the hospital. Nurses often had independent reporting systems to higher level health professionals and described experiences where the absence of doctors had been noticed only hours after they were due to attend work, if at all. They stated that often, there was a complete lack of structure in absence reporting among senior staff. With regular use of bank, temporary and contract staff, an accurate overview of absentee levels and reasons for absence within the hospital was deemed challenging.

Thirdly, all participants discussed the current role of occupational health departments and opportunities for their involvement in a surveillance system of this kind. Participants criticised the lack of funding and resources supplied to these departments, which currently played a minor role in managing HCWs' health beyond initial employment screening. They felt occupational health departments would be well placed to manage symptom and personal health data and organise diagnostic testing, and were keen to see their function broaden, assisting with the management of acutely as well as chronically sick staff, and in particular those symptomatic in the work place. Further, they felt independent assessments of symptomatic staff by occupational health departments would make decisions to send staff home '*more valid' *and minimise anxieties and scepticism (appendix 5). In all countries, participants described the current practice for sick HCWs to visit their own general practice doctors, highlighting the potential inability for these to identify unusual symptoms in the first instance and make time-spatial links with other possible cases. There was consensus on the need to expand standard management and control guidelines of infectious disease outbreaks in the work place beyond those for gastrointestinal infections. However, with occupational health departments struggling with current functions, such as promoting and providing seasonal influenza vaccinations, participants lacked confidence in the ability of these departments to manage staff with acute infections and perform epidemiological investigations. HCWs' perceptions of knowledge and structural barriers are presented in appendix 5.

## Discussion

### Principal findings

HCWs identified risk factors for occupationally acquired infections to be exposure to undiagnosed infectious patients, especially in accident and emergency wards, break-down in infection control procedures, immuno-naïvety and symptomatic colleagues. A further concern was a lack of monitoring and guidelines allowing health professionals to work whilst infectious. All participants felt HCWs' health needs were perceived as of low importance and described feelings of anxiety when reporting sickness related absences which were at times received with scepticism. Staffing demands and loss of income were noted as pressures for symptomatic HCWs to come to work.

HCWs had mixed views on the feasibility and acceptability of monitoring their absences and symptom reports for the detection of unusual cases or clusters of cases of infectious disease. All felt they had experienced activities similar to those described in the suggested monitoring system during the recent pandemic. However, in the absence of a clearly perceived threat, issues concerning privacy, confidentiality and sensitivity were raised and, particularly in Germany, identified as barriers. Nevertheless, in the UK, Hungary and Greece, participants felt the disclosure of symptoms for monitoring would be appropriate, provided the rationale, purpose, effectiveness and efficiency of such a system could be demonstrated.

Beyond individual preferences for monitoring absence and disclosing symptoms, knowledge and structural barriers for early recognition of EIDs were recognised. The potential lack of suspicion and awareness among HCWs towards new infections was of concern as EIDs were perceived as rare events. Financial and labour resources required for implementation would impose a burden on already stretched services. The heterogeneity in absence monitoring across professions and lack of communication between working groups would hinder accurate data collection on absence. Furthermore, all participants criticised the lack of funding and minor role of occupational health departments in managing the health of hospital staff. Participants, especially in the UK, were keen for occupational health departments to increase their involvement and assume responsibility for staff with acute as well as chronic conditions. They felt occupational health departments maintained an independence making them well placed for the management of personal health data and symptomatic staff in the work place.

### Strengths and weaknesses

This is the first study to use a common methodology to examine HCWs' perceptions and attitudes in different European countries towards monitoring their absence and symptoms for the surveillance of EIDs. It is unique in exploring a diverse range of healthcare professionals' risk perceptions in relation to occupationally acquired infectious diseases and sensitivities around the disclosure of symptoms in light of the recent history of the emergence of new pathogens. The study has provided insight into knowledge gaps and structural barriers to the timely detection of newly circulating infections in hospital settings.

General limitations of this type of research are selection and response bias. The focus group participants may not be representative of healthcare worker and hospital populations and research in other settings may have produced different results. Group dynamics may have influenced individual participants' responses in a way perceived to be culturally desirable. Data from qualitative research is also generally at risk of decontextualisation and misinterpretation, perhaps more so in this case where the audio of four of six focus group sessions were back translated into English. Attempts were made to minimise this with the review of transcripts and analyses by the facilitators. Facilitators may have exhibited different interview techniques, potentially examining themes to varying extents, but they all had a common briefing and this approach permits identification of culturally specific factors. Not all EU countries were represented in this analysis and other cultural and structural differences may have generated different findings.

### Comparison with other studies

To the best of our knowledge, there are no previous reports of similar studies, particularly none that examined sensitivities around absence reporting and the disclosure of symptoms by HCWs. Some of the key findings here have been highlighted in the 2009 NHS Health and Well-Being Review Interim Report [14]. These include under-resourced occupational health services and uncertainty over the role and function of these departments (including the balance between supporting staff and managers), the issue of HCWs coming to work when feeling unwell, and the perception that senior managers or the employing organisations fail to take a positive interest in HCWs' health. Another study has also provided evidence that HCWs attend work with symptoms suggestive of communicable disease [15].

Similar to findings here, a study by Blake et al. found a loss in income was associated with the general working population's ability to comply with recommendations during an influenza pandemic in the US, resulting in symptomatic staff in the workplace [16]. A recent survey, also in the US, found that 55% of workers without sick pay compared to 37% with, would attend work with an infectious disease [17].

There is evidence to support HCWs' fears of the increased risk in accident and emergency and intensive care departments as studies had found H1N1 attack rates among staff highest in these departments [5,18]. Concepts for a surveillance system in this population could focus primarily in these departments where HCWs may have a higher invested interest. However, even during a pandemic, at varying World Health Organisation levels of alerts, it was difficult to motivate HCWs to take precautionary measures [19].

### Implications

The main findings from this study reveal that developing a European surveillance system for infectious diseases in HCWs using absence and symptom reports would need to overcome cultural and logistical barriers but would nonetheless probably be feasible if HCWs can be convinced that it has value. Differences in the perception of risk and need for monitoring will challenge any unified system. Economic and structural barriers for HCWs to manage their health, such as loss in income and staffing pressures will undermine the development of concepts for this type of surveillance, as it is based on encouragement and the responsibility of individual staff to report when unwell.

Grounds for variation in HCWs' perceptions on the appropriateness to ask to report symptoms seem to be cultural, with participants in Germany holding stronger views on the need to protect privacy and confidentiality. Any concepts developed for surveillance in this population will need to be sufficiently flexible to embrace a wide range of attitudes and practices. An example to circumvent barriers potentially predominantly in Germany may be to use aggregate data without identifiers and information on reasons for absence to highlight patterns. This concept has been explored in further detail in the REACT surveillance system framework model report [12].

There is a need for more education on infectious disease epidemiology among hospital staff and further work to be done to increase adherence to recommended infection control practices. EIDs may be perceived as rare events; however their occurrence can be catastrophic on numerous levels. Early detection and response is crucial for control on a global level. Infections in HCWs pose risks to patients [15] and HCWs need to feel protected to deliver an efficient and effective response, which public health experts must consider for preplanning response frameworks [3,20]. The resonance across countries in underfunded occupational health departments is worrying as is the poor understanding of the risk and consequences of nosocomial outbreaks.

Aside from the detection of newly circulating infections, there may be many additional benefits from monitoring absence and symptom reports of hospital staff. These data may be used to identify patterns of absence and their cause, highlight problem areas, provide transparency, pinpoint areas where infection control procedures break down and prevent sickness consequentially reducing costs. In the UK, sick leave currently costs the National Health Service (NHS) 10.3 million working days and £1.7 billion per year, with higher rates than anywhere else in the public or private sector [14]. Cutting sick leave even by a fraction could save the NHS millions of pounds, and may be achieved by increased vigilance and encouragement for symptomatic staff to stay home, reducing the spread of infection. Further, reducing the spread of infection will decrease the risk of the need to close wards which incurs huge opportunity costs for elective procedures as well as significant financial losses [21].

## Conclusions

Asking HCWs to report symptoms when absent from work with a suspected contagious illness may be feasible as a means of surveillance for EIDs in some settings. However, more fundamental issues need to be addressed for the further development of this concept. These include standardising a culture of safe healthcare with education and compliance in infection control, tackling staffing pressures, homogenising absence recording, reviewing sick pay entitlements and improving the understanding of nosocomial outbreaks. Pilot studies will need to be tailored to specific countries and hospitals to investigate how these initial concepts may be adapted.

## Competing interest statement

All authors declare that they have no competing interests.

## Authors' contributions

AA and GE designed the study, reviewed the literature, analysed the data and were responsible for writing the manuscript. FN, GT and MC assisted in coordinating the study. GE, GT, ES, TE, GP facilitated the focus group discussions in their respective countries. All authors commented on drafts of the manuscripts and approved the final version.

## Appendix 1. Topic guide sample questions

What different experiences do focus group discussion members have in terms of outbreaks of infectious disease in the hospital and how common is it for hospital staff members to contract infectious diseases at work?

What are your views on the need for more measures to protect healthcare workers from work associated infections? Discuss the pros and cons of various methods and how it may impact on behaviours and reporting.

What are your views towards an ongoing surveillance system monitoring absence and symptom reports of HCWs? Discuss views on practicality, simplicity, acceptability, adaptability to different departments, concerns, suitability for prevention of outbreaks and protection of staff. Is there a place for a surveillance system of this kind in your hospital?

How would you feel about describing symptoms to your manager? As a manager how would you feel about staff describing their symptoms to you?

How would you feel if this were to be implemented in your hospital? Would you be willing to participate in a pilot study?

## Appendix 2. Participants' views on risk factors for occupationally acquired infections

### Higher risk in A & E wards

(UK) "... around the time of the swine flu epidemic, I did feel a bit vulnerable at that stage because people would present to A&E and at that point, you don't know what they've come in with and then we get them the mask."

(Hungary) "...here [in emergency medicine] we investigate, send samples for testing, make a possible diagnosis of infectious illness, then get afraid that we may have been infected. THEN they put on a mask and gloves. "

(Greece) "Where an [ER] patient with an airborne-infectious disease is present and coughs, it makes up an infectious situation in the sitting room and in the transmission department until the patient has been assigned."

### Factors causing a break-down in infection control

(UK) "(Infection control may break down when) not having equipment ...not having the knowledge to know what to do and when to do it right."

(Hungary) "'In an emergency we have to save lives, and if a few bacteria or viruses are transmitted in the process so be it."

(Hungary) "In everyday, real life, there is not sufficient supply of protective gear. We had difficulties in dealing with the financial departments to try to increase this stock."

### Immuno-naïvety

(UK) "I'm new to infectious diseases so from January I've had a chest infection, an ear infection and throat infection."

### Low risk perception of EIDs

(Hungary) "I do not believe it [SARS] would just suddenly appear at an emergency department. Or if it was to suddenly appear nobody would know, and the patient would die."

### Current monitoring systems

(Hungary) "Hopefully, as with H1N1, there would be an outbreak in some other country, which would be followed by the setting up of a monitoring system."

(Germany) "An epidemic alarm plan has been established, for pandemics, ... the more the topic is discussed, the more aware each person becomes... for example in emergency admissions, we have inspected the premises and doors, which have to be plastered up in urgent cases [...] but I can't envisage it leading to any kind of constant surveillance system, I mean what would they be reporting the whole time?"

## Appendix 3. Healthcare workers' perception of illness and sickness-related absence

### Barriers towards reporting: Perceptions of HCWs' health

(Hungary): "Staff health has a lower importance, but this obviously depends on the given staff (occupational) health unit, how well organised it is, how much the employees can rely on it."

(UK) "Is that because we all are a bit unsympathetic to our own needs as staff? We're very sympathetic to patients but probably to our own staff, we're not very good, we don't probably listen to them and think...like I've just said we think they're lying."

### Barriers towards being absent from work when unwell: Staffing pressures

(Hungary) "...they may be more concerned that there will be nobody to replace them, and there will be inadequate staffing."

(UK) "But I think the issue with ward managers and that grey area is that if you send somebody home who are you going to replace? That member of staff, there's never a spare person."

(UK) "...and then we curse them (the absent HCWs) because they've left you in a position where you've got no staff again."

### Sick leave

(Hungary) "As long as sick leave does not pay a hundred percent of their usual salary, which is often the case, they will continue working or take some paid leave so they would not suffer a loss of income. Even in cases of MRSA..."

(Hungary) "Self-employed staff are reluctant to report (sick)...following an illness with diarrhoea they should not resume work for 48 hours as they are still spreading the infection, but they often say they 'do not care' because they are not paid ..."

(Germany) "There is this fact: Germany's employees do not take sickies, because the economy is now worsening and it tends to make people get their acts together..."

### Expectations and habits for HCWs to work with infectious disease symptoms

(UK) "With norovirus some people didn't want to go off duty so sometimes they could have been working and it was symptomatic. ...you had to physically make them go off duty."

(UK) "That happened with the flu though; doctors were the worst weren't they? You'd find doctors walking round sniffing and sneezing and have you been like that, you need to go home."

(UK) "It's a bit ironic because the advice we gave to relatives was if you had any symptoms, if you had a cold, if you had anything then you shouldn't come and visit. ... Yet I suppose as managers I probably expected my staff to come in if they were slightly under the weather."

(Greece) "...we all have the tendency to come to work even if we don't feel well."

(Greece) "For the medical practitioners, it is their symptom of high fever which would keep him/her away from work. If gastroenteritis, as mentioned, is not at a risk stage, then they could technically function as long as they don't have diarrhoea 4-5 times per day, or acute pain."

## Appendix 4. Healthcare workers' willingness to report infectious disease symptoms for surveillance purposes

### Factors contributing to willingness to report symptoms

#### Genuine illness

(UK) "...If you're genuinely ill and you've got nothing to hide then I'd be comfortable, it wouldn't bother me, I'd tell everything."

#### Understanding the rationale

(UK) "I think most people would be happy if they knew there was a risk, if you could tell them why you're asking the questions."

(UK)" If all the staff were educated on that, like if you've got D&V, please report it, surely they should take a little bit of ownership and report it."

#### Informal reporting

(Hungary) " I feel that in case of a healthcare worker [...] if it is anything acute they will call in and say 'I have diarrhoea' or 'I have a fever' 'I can not get out of bed'."

(Germany) "Because everyone knows each other on the wards and there are many friends there, it's not just 'I am ill now' but 'I feel lousy'. 'I am retching all the time, and vomiting' and then you get the next person who says 'I also had that last week', who happened to have the same symptoms, so I think, it is informally but frequently fed back like that."

### Reluctance to being monitored and consequences for the infected HCW

(Germany) "Well, I certainly wouldn't tell you what kind of symptoms I have. And even if I did, it would only be informally. Peter's got diarrhoea just like I had last week. If the whole process were structured, it would be a refusal."

(Germany) "In this period, the question has also been raised, what do we do with hepatitis positive staff, the idea that you would out them is almost a matter affecting the employment contract."

### Factors affecting the validity of self reported symptom data

#### Accuracy of staff reports

(UK) "...people will ring up and say I can't come in, I've got abdo pain, but really, they've just split up with their husband or their wife or something and that's something that then comes out when you're speaking to your line manager or something."

#### Accuracy of data collected

(UK) "When you take phone calls off the staff, they just say they've got diarrhoea, some are embarrassed really so you don't really go into depth. Whoever takes the phone call, it's not necessarily yours, it could be whoever's on charge of that shift and whether they pass the message on correctly or do they ask them why they're off sick, some people don't, they just go 'oh okay' and put the phone down."

(Greece) "Most of the times when (sick leave) notes are composed they are not real, so this inflicts distrustfulness and false information within the data given on files. When they received the note they are quite healthy."

### Barriers to data collection

#### Shift patterns

(Greece) "...there is a way so that the absent shifts are covered in [a] different way where [the] illness is not declared."

### Confidentiality, data protection and legislation

(Hungary)" Well I don't see that [the proposed monitoring system] as so positive. I can't imagine it working well, because I think in many areas, the need for data protection and protection of identity of any kinds of diagnoses is much higher [...]"

(Germany) "...but certainly, the workers' council would be the key issue and confidentiality would make it very difficult in Germany to find a regulation, which would also be acceptable from a data protection perspective, the question always looms in the background, which disadvantages would the employee, admitting to such a thing in this context, namely having such and such an illness, be subject to, perhaps suspended from service or not allowed to work with patients any longer."

### Perceived benefits of monitoring

#### Identify staff absenteeism

(UK) "...you might find the person that's on a certain ward, every time they're put on that ward, they don't like it, they're off. So you can track like that, you know, what's the problem."

### Improve infection control procedures

(Hungary)"It may be also useful to monitor appropriate hygiene, because if a certain ward has an increased number of an illness, there may be problems with staff hygiene."

(Hungary) "I feel that if it would lead to more education about prevention, and it would be established with good intentions, then it would just be to the benefit of the nursing staff."

### Prevent spread of infection

(Hungary) "Had the case been reported after the first two or three illnesses, we could have advised on preventative measures to be taken and the entire thing would not have spiralled so far. I do think that proper reporting and monitoring has an important role in reducing the length and the number of people affected by an epidemic."

## Appendix 5. Barriers for the timely recognition of outbreaks in the hospital environment

### Lack of knowledge

(Hungary) "Infection control is not an incorporated subject in doctor training, and old habits and bad habits are much harder to change. It would be of utmost importance to have infection control taught to medical students and specialist trainees. To get an early concept of these matters."

### Heterogeneity in absence monitoring

#### Differences in reporting among staff

(Hungary)" You have been talking about nursing unit managers and nursing staff, but these people are more aware of infectious matters and I always felt the 'weak link' was the doctors... Yes. Totally. Yes. Up to the point when they have an epidemic that completely stops the ward from functioning, this is their nightmare"

### Staff based at multiple sites

(Hungary)" In the case of self-employed doctors who work at several hospitals, it is virtually impossible to follow MRSA for example, because if during an outbreak we test doctors as well and it turns out their results are positive, I am convinced that 99 percent of them will not report to their other employers that they are carriers of MRSA: Because this would lead to a decrease in income."

### Lack of absence reporting mechanism

(Greece) "I have begun to file the absences of the staff under order of the Administration, and there is not one day of absence recorded by the medical practitioners. I believe that between us we certainly know of someone who has been ill, but because of our relationships with one another, the medics don't really take any sickness leave. They inform you, but they aren't declared and recorded."

(Hungary) ".. for agencies who send nursing staff [...] if one person is ill, they will just send somebody else and nobody will know that the ill person has something infectious."

### Financial and resource burdens

#### Financial constraints

(Hungary) "On the whole we see no obstacle to Hungary joining any unified European system, but the resources would not be made for this to happen. Isolation of patients or personal protective equipment is quite expensive."

### Lack of skill and resources in occupational health departments

(Hungary) "We also have a staff health service, with such minimal number of people involved that it is shameful, and they themselves do not really try to break out of their limitations, with their goal being the achievement of routine checks being completed and that is about it. We have turned to them on one occasion to please make a report of the illness of workers in the hospital kitchen and even that lead to complete chaos as to what they need to do and how. They do not really care about infectious diseases. When there is a mention of contagious illness the first thing they do is call us."

## Pre-publication history

The pre-publication history for this paper can be accessed here:

http://www.biomedcentral.com/1471-2458/11/541/prepub
